# Arduous implementation: Does the Normalisation Process Model explain why it's so difficult to embed decision support technologies for patients in routine clinical practice

**DOI:** 10.1186/1748-5908-3-57

**Published:** 2008-12-31

**Authors:** Glyn Elwyn, France Légaré, Trudy van der Weijden, Adrian Edwards, Carl May

**Affiliations:** 1 Department of Primary Care and Public Health, School of Medicine, Cardiff University, Heath Park, CF14 4YS, UK; 2 Department of Family Medicine, Université Laval, Centre Hospitalier Universitaire de Québec, Hôpital St-François d'Assise10 Rue Espinay, Québec, G1L 3L5, Canada; 3 Department of General Practice, School for Primary Care and Public Health (Caphri), Maastricht University, PO Box 616, 6200 MD Maastricht, Netherlands; 4 Institute of Health and Society, Newcastle University, 21 Claremont Place, Newcastle upon Tyne, NE2 4AA, UK

## Abstract

**Background:**

Decision support technologies (DSTs, also known as decision aids) help patients and professionals take part in collaborative decision-making processes. Trials have shown favorable impacts on patient knowledge, satisfaction, decisional conflict and confidence. However, they have not become routinely embedded in health care settings. Few studies have approached this issue using a theoretical framework. We explained problems of implementing DSTs using the Normalization Process Model, a conceptual model that focuses attention on how complex interventions become routinely embedded in practice.

**Methods:**

The Normalization Process Model was used as the basis of conceptual analysis of the outcomes of previous primary research and reviews. Using a virtual working environment we applied the model and its main concepts to examine: the 'workability' of DSTs in professional-patient interactions; how DSTs affect knowledge relations between their users; how DSTs impact on users' skills and performance; and the impact of DSTs on the allocation of organizational resources.

**Results:**

A conceptual analysis using the Normalization Process Model provided insight on implementation problems for DSTs in routine settings. Current research focuses mainly on the interactional workability of these technologies, but factors related to divisions of labor and health care, and the organizational contexts in which DSTs are used, are poorly described and understood.

**Conclusion:**

The model successfully provided a framework for helping to identify factors that promote and inhibit the implementation of DSTs in healthcare and gave us insights into factors influencing the introduction of new technologies into contexts where negotiations are characterized by asymmetries of power and knowledge. Future research and development on the deployment of DSTs needs to take a more holistic approach and give emphasis to the structural conditions and social norms in which these technologies are enacted.

## Background

There is increasing interest in interventions that help patients become involved in decision-making about healthcare choices. One set of such interventions are known as 'decision aids', interventions that provide decision makers with information about the nature and probabilities of options and their attributes, assume that a deliberate choice is necessary, and often, though not always, provide methods to deliberate or clarify 'values' [1]. These exist in a number of formats (paper-based, video, and web) and there are many ways in which they can be used in practice. They may be given to patients before consultations or made available for use during or after consultations with health professionals, either with the professional who is directly dealing with the patient or by asking the patient to receive guidance by another health professional. Therefore, there are a number of ways in which interactions using these interventions can take place that involve different settings and different professional groups. These interventions have a proliferating number of names including: 'patient decision aids' and 'decision support tools' among others. In this paper, we use the term decision support technologies (DSTs) to provide a generic description and to make the connection to the widely recognized interest in health technology assessment. In this context, O'Connor *et al*. [1] have defined DSTs as interventions:

'designed to help people make specific, deliberate choices among options (including the status quo) by providing information about the options and outcomes (e.g., benefits, harms) in sufficient detail that an individual could personally judge their value.'

These technologies may include:

'information on the clinical condition; outcome probabilities tailored to personal risk factors; an explicit values clarification exercise (e.g., a relevance chart, utility assessments of probable outcome states, a weigh scale); descriptions of others' experiences; and guidance in the steps of decision-making and communicating with others.' [1].

There are now reports of large numbers of DSTs. A systematic review has been conducted [1], an inventory of such interventions is available, and a system to assess their quality is also being developed [2]. Although clinical trials seem to show that DSTs are useful in clinical practice, it is also clear that these technologies – and the shared decision-making approach which underpins their use – are not being widely adopted by health care professionals [3,4]. Shared decision-making is used here to describe an approach of actively involving patients in making health care decisions. The approach assumes information provision and the existence of equipoise (legitimate viable options) [5], so that patients, when informed may choose to be involved to the 'extent they prefer' [5], recognizing that some people prefer others, such as health care professionals, to take decisions on their behalf.

Although numerous reviews have considered how best to implement clinical guidelines and other forms of evidence or evidence-based practice, few studies have examined the difficulty of introducing DSTs into routine practice in any depth. In those that have, a 'many barriers' argument has been an important explanation, such as the report by Holmes-Rovner *et al*. of a study to determine the feasibility of DSTs in fee-for-service hospital systems including physicians' offices and in-patient facilities [6]. Holmes-Rovner *et al*. reported that the key obstacle was time pressure, although the authors also raise the possibility that this may not have been the only factor. They conclude that, to be successful, implementation processes would have to include system changes, such as the integration of DSTs into an informed consent process, or incentives such as payer negotiated requirements (where shared decision processes are assumed to be quality indicators), or reimbursement to professionals who make shared decision programs available to patients. Gravel and Légaré's systematic review revealed a taxonomy of barriers, including time constraints and lack of applicability to patient characteristics and to clinical situation [7]. Such factors draw attention to individualized problems of employing DSTs, and it is increasingly recognized that the successful adoption of interventions depends on more complex interactions than one of overcoming barriers [8,9].

We argue that a 'many barriers' explanation is insufficient and that a more holistic perspective is necessary. Existing theoretical models often focus on implementation and adoption of new technologies in terms of individual behavioral change [10-12], or organizational diffusion [13-15], rather than in terms of the work of using DSTs in practice. This is a core, but under-recognized, problem for DST researchers: the language of adoption and implementation of innovations dominates policy and practice debates about employing DSTs in clinical practice to the exclusion of considerations of their workability and integration for users. If we wish to understand why DSTs seem not to be operationalized by professionals, even when they are widely diffused and available, then it is their everyday embedding in clinical practice – rather than innovation and adoption by healthcare providers – that should be the focus of our attention. In this paper we have used a theoretical framework – the Normalization Process Model (NPM) [16-18] – to explain factors [6,7,19-27] that promote and inhibit the implementation of DSTs in routine practice settings.

### The Normalization Process Model

The NPM developed by May and colleagues is a theoretical model that focuses attention on factors that have been empirically demonstrated to affect the implementation and integration of complex interventions in healthcare [28]. See Table 1 for definitions of its constructs and dimensions. It is intended to facilitate understanding from a process evaluation perspective, and has been used across a range of contexts [29-33]. Normalization is defined as the routine embedding of a complex intervention in healthcare work [16], and the NPM offers a robust structure for investigating the collective work that leads (or not) to this. The NPM is structured as follows.

**Table 1:** Definitions of constructs and dimensions of the Normalization Process Model applied to Decision Support Technologies

**NPM Constructs**	**NPM Dimensions**	
**Interactional Workability: **People operationalize a DST when they engage in work that characterized by specific patterns of conduct (congruence), and expectations about their outcomes (disposal).	**Congruence **requires shared expectations of the normal conduct and purpose of the clinical encounter; the roles of participants; and the legitimacy of shared decision-making.	**Disposal **of participants' problems requires agreement about the meaning and consequences of the shared decision; and expectations of the goals and possible outcomes of the clinical encounter

**Relational Integration **People organize a DST through working to share knowledge and practice (accountability), and beliefs about its value and meaning (confidence).	**Accountability **requires agreement about the knowledge and expertise that underpins the shared decision; beliefs about their validity and significance; and agreement about the interpretive contribution of participants.	**Confidence **requires agreement about the authority and credibility of the knowledge and expertise through which the shared decision is framed; or beliefs about the utility of this knowledge and the criteria by which it is evaluated.

**Skill-set workability **People distribute the work connected to mobilizing a DTS according to specific formal or informal roles (allocation), and evaluated by reference to shared beliefs about action (performance).	**Allocation **requires agreement about the assignment of shared decision-making tasks to participants; beliefs about the ownership and appraisal of the skills; the distribution of resources and rewards; and mechanisms to record participation.	**Performance **requires agreement about the content of shared decision-making tasks assigned to participants; shared beliefs about the boundaries of their responsibility; and mechanisms to decide the degree of autonomy available to them.

**Contextual Integration **People enact a DST by working to assign the necessary intellectual property, personnel, and material resources (execution); and to seek to link it to its operational contexts by sustaining the allocation of these resources (realization).	**Execution **is made possible by participants' agreement about distributing responsibility for the conduct of shared decision-making; policies for allocating intellectual and capital resources to participants; and mechanisms for linking participation to organizational structures.	**Realization **is made possible by participants' agreement about the value of shared decision-making; policies about the procurement and delivery of personnel and equipment; and mechanisms for modifying organizational objectives.

### Context

Implementation processes are composed of chains of interactions in which a complex intervention (a new or modified way of thinking, acting upon, or organizing practice) is made coherent and enacted in a healthcare setting. Implementation processes are managed and 'owned' through behaviors that denote cognitive participation by healthcare professionals and other personnel, including patients.

### Collective Action

A complex intervention is enacted through different kinds of interactional and material work. This work may be highly structured (enacting a research protocol, for example), or diffuse (in operationalizing a policy decision in a large organization). This work is located in the endogenous or immediate conditions of encounters between people using the intervention, and the exogenous conditions that structure these encounters.

In their immediate conditions of practice, people operationalize a complex intervention when they engage in co-operative interactions that are characterized by specific patterns of conduct (congruence), and expectations about their outcomes (disposal). The potential operationalization of a complex intervention is determined by its 'interactional workability'. People organize a complex intervention through shared knowledge and practice (accountability), and beliefs about its value and meaning (confidence) within organizational networks. The potential of a complex intervention to be embedded in a network is determined by its 'relational integration'.

In the exogenous conditions that structure encounters between participants in a complex intervention, work is distributed according to specific formal or informal roles (allocation), and evaluated by reference to shared beliefs about action (performance). The distribution of work connected with a complex intervention is determined by its potential for 'skill set workability' within a division of labor. People enact it by drawing on their capacity to assign the necessary intellectual property, personnel, and material resources (execution); and to seek to link it to its operational contexts by sustaining the allocation of these resources (realization). The capacity of people to participate in or with a complex intervention is determined by its potential for 'contextual integration' into the specific setting.

### Reflexive Monitoring

Patterns of collective action and their outcomes are continuously evaluated by participants in implementation processes, and the formality and intensity of this monitoring indicates the nature of cognitive participation and collective action. Formal patterns of monitoring (for example, clinical trials) focus attention on normative elements of implementation (measuring them against ideas about how things ought to be [34]), rather than the conventions (how things are worked out in practice [35]) of social relations and processes upon which informal patterns of monitoring are focused. The shift from formal to informal appraisal by participants is an important signal of the routine embedding of a complex intervention.

Set out in this way, the model offers a simplifying structure for understanding three things: the relationships between a complex intervention and the context in which it is implemented; the processes by which implementation proceeds, including interactions between people, technologies, and organizational structures, and the work that proceeds from these; and a process-oriented assessment of outcome that also considers the potential and actual workability and integration of a complex intervention as accomplishments of its users.

## Methods

Our purpose in this study was not to test the model by experiment or systematic review. Instead, GE, FL, AE and TvdW (physicians and researchers in the DST field and in implementation studies) wished to decide whether the NPM (which at that stage was newly developed) was of value in understanding the difficulties encountered in getting DSTs embedded into practice. They collaborated with CRM (a sociologist, and author of the NPM) to test the conceptual adequacy of the model. Between February and June 2007 we used a collaborative online spreadsheet (a tool provided by Google) as a virtual laboratory for a series of thought experiments [36]. Although there are many different categories, this method has a long tradition [37]. In essence, these experiments represent patterned ways of thinking that allow new insights, including analysis, explanation, or prediction. In this study, a thought experiment is used to examine a novel model and test its propositions, against evidence from empirical studies, where available, and if absent, to see where gaps exist. These were analytic processes in which we operationalized NPM and examined how the model applied to the work of implementing DSTs. Conducting these analyses involved three discrete ways of working. These developed organically over time: beginning by asking whether the NPM was relevant to research on shared decision-making (a process of clarifying and explaining the model), and then whether its constructs mapped on to the results of existing research (reading the model against our own work and that of others [6,7,19-27]), and finally, as noted above, asking whether the NPM helped to explain those factors that promote or inhibit the implementation of DSTs in practice and in addition, considering where the model needed to be developed. The NPM is a general model but, like all such models, requires interpretation according to the specific features of the question which it is addressed. In Table 1, we show how the constructs and dimensions of the general model are interpreted in understanding problems of implementation and integration of DSTs.

First, participants drew together data from several different but related bodies of knowledge (including participants' observation and experience, formal evaluations, and other theoretical literature) of shared decision-making (as a social context) and DSTs (as actors in that context), in which we qualitatively manipulated data composed of materials derived from systematic reviews and primary research studies [6,7,19-27]. Data drawn from these sources were used to populate the cells of the spreadsheet with three kinds of attributions. For each construct we provided: general theoretical statements (describing the model); empirical generalizations drawn about DSTs (mainly derived from reviews); and specific attributions about the workability and integration of DSTs into practice (drawn from primary research). These formed statements about what was already known and understood about both DSTs and shared decision-making. We then applied the NPM to the explanation of these statements, asking what would be the case if 'a state of affairs described in some imaginary scenario were actual' [38]. In this work, participants sought to build an explanation of the phenomena in question by applying the propositions of the NPM. Finally, the products of this work were organized as structured explanations of the collective work involved in operationalizing DSTs, with and without shared decision-making processes.

## Results and Discussion

**Figure 1:**
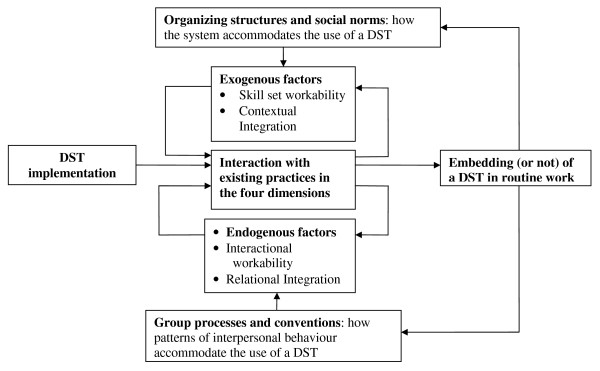
**Normalisation Process Model applied to the implementation of a DST**.

Applying the NPM enabled us to define the problems of routine embedding of DSTs in clinical practice in a structured parsimonious way. The NPM draws attention to ways of working towards shared decision-making rather than to the 'technology' as a vehicle for information delivery. It forms a framework for the analysis and presentation of the results of our work: Figure 1 provides an overview of the model applied to the implementation of DSTs.

### Endogenous factors that affect the implementation of DSTs

Our analysis forced us to acknowledge the importance of other stakeholders. In Table 2 we identify the work that different actors need to do around the implementation of DSTs in the clinical encounter as these are suggested by existing research; in focusing on endogenous factors, our analysis also revealed that the existing research literature is unbalanced. It gives primacy to interactional factors found in the consultation. This reflects the 'many barriers' approach to understanding DSTs and other technologies, in which research has focused on the interactional and technical problems that physicians say intervene to make shared decision-making difficult in clinical practice. Clinicians' power to define their knowledge and professional interests in 'good' communications are central to this. There is now an abundant body of literature that focuses on verbal interaction between professionals and patients [39]. But the business of interaction is by no means the whole problem: the knowledge that underpins professional-patient interactions is also key. The credibility, confidence, and accountability frames of the professional network are typically oriented to expert-led decision-making rather than on the facilitation of preference-sensitive decision-making by patients [40]. In this context, professional 'resistance' to DSTs and shared decision-making in this context reflects the contest between new ways of working and existing normalized patterns of working that are reinforced by training, peer work patterns, and the expectations set up by prior encounters set in a tradition of practice.

**Table 2:** Endogenous factors that promote or inhibit the implementation of DSTs

**User groups**	**Physicians**	**Patients**	**Managers**
**Interactional Workability**	▪ Enrolling patients in shared decision-making	▪ Concept of shared decision-making	▪ Ensuring efficient and safe interactions.
	▪ Making DST available	▪ New role as participant	
	▪ Integrating DST in the consultation	▪ Cognitive engagement with DST	
	▪ Managing time to process patients	▪ Understanding and assessing outcomes	
	▪ Managing patients who do not enter into shared decision-making	▪ Decisional responsibility	

**Relational Integration**	▪ Linking DST to evidence base	▪ Making sense of clinical knowledge	▪ Assessing the value of evidence
	▪ Confidence in applicability to individual patients.	▪ Agenda setting over treatment outcomes	▪ Understanding professional engagement
	▪ Matching clinical evidence with patient knowledge		▪ Defining and evaluating 'best practice'
	▪ Deciding on patients' accountability for engaging with DSTs		
	▪ Dealing with safety and liability.		

Literature that focuses on the consultation seems to indicate that barriers to the use of DSTs are dominant in everyday practice [7]. If we use the NPM to frame a 'many barriers' approach, then we can argue that DSTs lack congruence with existing patterns of professional-patient interaction, and because they do not necessarily assist disposal. DSTs introduce core problems of confidence and legitimacy in their relationships with patients, and raise questions about who should be allocated such work and the skills that they need. A tension therefore exists – a difficulty of 'communication among different people's perceptual universes, such as those between therapist and client' [41] – that is of central importance to the interactional conduct of shared decision-making. However, there is a deeper problem at issue here. As Table 2 shows, the factors that we identified in mapping the NPM onto existing research suggest that there is a fundamental difference in the ways that the research literature identifies the work that goes into operationalizing a DST in practice. Put simply, professional and patient are not seen to be doing the same work.

The problem of different accountability frameworks is important. DSTs are designed and predicated on the assumption that involving patients in decision-making is a 'fundamental good' and part of best practice. It may be that although at policy levels many health care systems espouse the values of respecting patient choice and autonomy, the organizational norms at face-to-face encounter levels favour a different set of values, aligned with getting work done efficiently. DSTs are also predicated on the ethos of being explicit about uncertainty, on the need to examine preferences, and provide information for patients so that they can participate fully in decision-making processes. Again, this ethos is not at all ubiquitous in practice settings. When we map patient work against interactional workability, we note a number of key differences – notably they are expected to accept new roles, undertake more cognitive work (understand risks and probabilities), interact with technologies, and accept decisional responsibility [20]. Moreover, there also exists the ethical problem of insisting that patients accept decisional responsibility. The interactional struggle to secure that patients accept decisional responsibility is often problematic, given uncertain clinical outcomes, and when insisting on the transfer of such responsibility may cause distress – the problem of abandonment [42] and the difficult of mandatory versus optional autonomy [43,44].

### Exogenous factors that affect the implementation of DSTs

The NPM focuses attention on more than the interactional and relational constraints that affect implementation. Table 3 is interesting because it emphasizes the structural work that needs to be carried out to implement DSTs. This table also shows how research that focuses on clinicians – because they are seen as the users of DSTs – has the effect of concealing central problems of how work is organized, allocated, and resourced in practice.

**Table 3:** Exogenous factors that promote or inhibit the implementation of DSTs

**User groups**	**Physicians**	**Patients**	**Managers**
**Skill-set workability**	▪ Delegating to autonomous patients	▪ Skills for participation	▪ Specification of roles and competencies
	▪ Communicating clinical decisions and risks	▪ Accepting delegated clinical decisions	▪ Definition of standard operating procedures and job descriptions.
	▪ Identifying appropriate professional roles for DST delivery	▪ Gaining competence	▪ Defining decisions to meet organizational goals
	▪ Delegating to other professionals		
	▪ Identifying and evaluating competencies		
	▪ Defining boundaries between determinate and indeterminate decision-making		

**Contextual Integration**	▪ Allocating physical media		▪ Managing allocation decisions
	▪ Allocating time		▪ Organizing protocols
	▪ Appraising value		▪ Controlling budgets
	▪ Negotiating with managers		▪ Managing professional autonomy
	▪ Managing medico-legal concerns.		▪ Managing patient choice

Service managers' work on allocating and organizing resources at the meso-level has an impact on the micro-level encounter of the shared decision [39]. They are also accountable for public access to healthcare and the safety of new technologies. The micro-levels of professional practice where interactional workability is tested have not traditionally been areas in which the managerial gaze has been welcomed [45]. The manager's perspective, however, is also one in which deeply normalized patterns of interactional conduct are a problem because they retard attempts to make health services more responsive. There is no doubt that there is a trend to manage clinical interactions and that they are increasingly governed by external corporate forces [46], for example through frameworks for 'quality of care' and the use of protocols and guidelines.

However, managers are interested in efficiency. Health care service provision is normally measured by capacity and maximizing workflows. DSTs, however, aim to increase the patient-centered nature of interactions. DSTs do not increase the flexibility of workflows but explicitly promote informed choice, involvement in decision-making [47], satisfaction with decision-making [48], decision quality [49], match with values, low conflict [50], and decreased decision regret [51], and are aligned with efficient or high throughput service models. DSTs would confer value to a health system that had oriented its metrics to these patient-centred outcomes, but, as currently operationalised, they are at odds with the prevailing organizing social norms and metrics. Enhancing their contextual integration by demonstrating that they confer added value to healthcare outcomes may be a key step – but we contend that this will depend, critically, on how performance is measured. Once again, there are fundamental differences between the ways that different groups are assumed by the literature to engage with exogenous factors. The most important of these is how little is known about how DSTs affect patients. The assumption throughout is that DSTs matter as part of the consultation, but this may overestimate the importance of the clinical encounter in determining how patients respond to shared decision-making and DSTs. We do not know.

## Conclusion

Our contention is that the NPM helps us understand why it is so difficult to implement DSTs into practice and acts here as an explanatory framework. We wish to proceed to work that can test whether the model can also be predictive, although we are cautious about claiming power to foresee the outcome of processes characterized by complexity and emergence. We sought to develop and refine the NPM through a concept analysis approach. We did not systematically review literature or conduct secondary analysis of existing data sets. The weakness of the study is therefore that it relies on interpretive analysis rather than prospective and structured collection and analysis of new data or secondary analysis of already existing data. However, we were able to draw on a wide variety of work: including recent and highly relevant systematic reviews, primary studies, and theoretical studies we have individually and collectively undertaken. Our conceptual analysis therefore drew on our own knowledge of the field as well as on recent reviews. We contend that a further strength of this analysis was that one of the authors (CRM) was responsible for the development of the theoretical model, but that we balanced his defense of the model by involving expertise in implementation research, shared decision-making, and in the development and assessment of DSTs [2,52-54].

Despite these limits on our work, mapping the results of key studies and reviews against the NPM led us to question the 'many barriers' argument in favor of one that is aligned to the factors that support 'normalization'. From the perspective of a health professional, the informed choice and shared decision-making that provides the rationale for using DSTs is not universally accepted as the basis for medical practice. Indeed, there is substantial evidence that health professionals find it difficult to practice according to the requirements of patient-centered practice, and we have empirical evidence that they are reluctant to involve patients in decisions [55-57], and find it difficult to use DSTs [58]. One reason may be that professionals' and patients' contributions to shared decision-making and the use of DSTs may need to be rethought in terms of 'work' rather than 'knowledge'. Further research is needed to investigate this hypothesis.

One of the main insights gained by applying the NPM was the need to consider its propositions from the perspective of different actors, particularly when the intervention is an inherent component of interactions between the actors. We also gained insight into the exogenous factors that impact on the micro-interaction, and so gained a much broader understanding of the elements that need to be aligned to enhance implementation strategies. When coupled with the difficulty of integrating DSTs into workflows [59], we have noted that, when placed against the norms of existing practice, DSTs seem to lack interactional workability. However, we have pointed to the ways that the research literature focuses on the perceived interactional conduct of shared decision-making, and the use of DSTs at the expense of other areas of their implementation. The assumption that 'many barriers' operate to exclude DSTs from the consultation may be wrong. It may be more important to look from a systems perspective at the ways in which the work of different participants is defined and organized, and by whom this is done. We know a great deal about professional-patient interaction in the consultation, but much less about other important factors.

There are good reasons for wanting to attend to this wider framework of analysis. For example, let us imagine a context where professionals are required to accomplish shared decision-making (or perhaps rather to involve patients in decision-making to the extent of their preferences). Professionals are monitored for their ability to accomplish these specific tasks, and they are applauded by their colleagues for accomplishing them. Let us further imagine a context where the skills of using DSTs are taught and evaluated, and the DST and work of engaging patients are part of the existing guidelines and embedded in the multi-disciplinary culture of the clinic – information exchange is initiated at entry and is an iterative process because patients are asked to assess their experience in the clinic by their recall of these processes. Health professionals and the managers are dependent on the presence of DSTs to accomplish their work – without them they could not achieve or realize their performance metrics – the percentage of patients who make or who are offered to make informed preference sensitive decisions. In this imagined clinic, all four propositions of the NPM are being met – the main change is the goal being set and a commitment to assess achievement against it [60]. Complex interventions perhaps – but a few simple rules could help align professional practice with the objectives and support the normalization of shared decision-making and DSTs [61]. The introduction of legislation in the Netherlands for example [62], and in 2007, in the state of Washington in the US, endorsing the benefits of shared decision-making processes and patient decision support technology is a signal that contextual influences are changing.

## Competing interests

The authors declare that they have no competing interests.

## Authors' contributions

GE initiated the study and all authors collaborated in the data collection, analysis and drafting of the manuscript.
